# A Rare Case of Gout and Pseudogout Occurring in the Same Joint

**DOI:** 10.5704/MOJ.1903.011

**Published:** 2019-03

**Authors:** YH Wang, SWL Ho

**Affiliations:** Department of Orthopaedic Surgery, Tan Tock Seng Hospital, Singapore

**Keywords:** gout, pseudogout, crystals, concomitant

## Abstract

Pseudogout and gout are common types of inflammatory joint disease in the elderly. However, the existence of both in a single joint in a patient is relatively rare. This case report describes an interesting case of a 42-year old man who presented with simultaneous gout and pseudogout in the knee joint, diagnosed via polarised light microscopy. There was no radiographic evidence of pseudogout. This case report serves to illustrate the need to actively exclude concomitant pseudogout, especially in patients suffering from recurrent attacks of gout.

## Introduction

Pseudogout and gout are common types of inflammatory joint disease in the elderly^1^. However, the existence of both gout and pseudogout in a single joint in a patient is relatively rare^2-4^. Furthermore, the concomitant diagnosis of pseudogout has largely been based on the radiological findings of calcification^3^, with few reports in the literature describing the specific microscopic findings of both types of crystals in the same joint^2,4^. This case report serves to highlight the possibility of both pathologies occurring simultaneously in the same joint.

## Case Report

A 42-year old gentleman presented to the Emergency Department with a 2-day history of right knee swelling and pain. It was of spontaneous onset, with no history of trauma. He reported recurrent episodes of right knee swelling over the last two years. Previous right knee arthrocentesis revealed negatively birefringent crystals for which he was treated with colchicine. Other significant medical history included right knee grade 3 chondromalacia patella, asthma and ischemic heart disease.

**Fig. 1:**
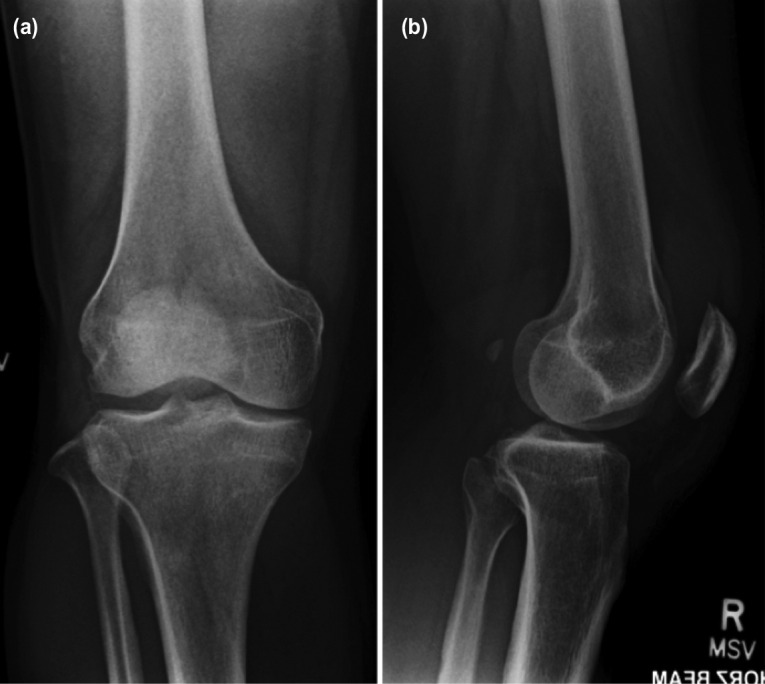
(a) Antero-posterior and (b) lateral radiographs of the right knee did not reveal any radiographic signs of chondrocalcinosis.

**Fig. 2:**
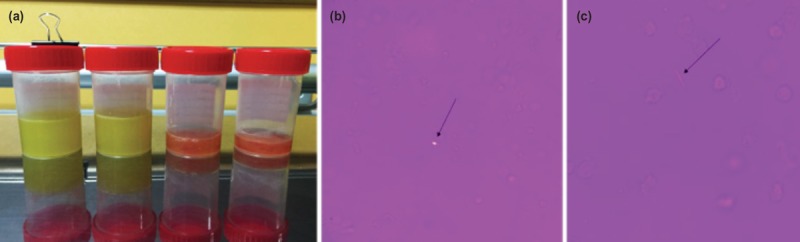
(a) Knee joint aspiration showed turbid fluid (all the four bottles are from the same knee). (b) Gout crystal (strongly negative birefringent needle-like crystals). (c) Pseudogout crystal (weakly positive birefringent rhomboid shaped crystals).

Physical examination revealed warm, erythematous right knee joint with moderate effusion. This was associated with mild medial joint line tenderness. The range of motion of the right knee was 10° to 40° limited by pain. Biochemical investigations showed a raised total white blood cell count of 10.6x10^9/L (4.0-9.6x10^9/L), erythrocyte sedimentation rate 28 mm/hr (1-10mm/hr), C-reactive protein 60.5 mg/L (0.0-5.0mg/L) and uric acid level of 575 umol/L (250-550umol/L). Radiographs of the right knee revealed degenerative changes, with mild narrowing of the medial compartment (Fig. 1). No calcification within the joint was noted. A joint aspiration was subsequently performed and turbid straw-coloured was aspirated. Fluid analysis revealed nucleated cell levels of 19,900 cells/uL (reference range <200 cells/ul), and a neutrophil percentage of 91%. The fluid gram stain and cultures were negative. Microscopic analysis of the fluid revealed scanty crystals that were both negative and positive birefringence (Fig. 2), which was consistent with the diagnosis of gout and pseudogout respectively.

The patient was treated with physiotherapy and colchicine. Non-steroidal anti-inflammatory medication was not started due to a history of ischaemic heart disease and active asthma. The patient was prescribed paracetamol and tramadol for analgesia. Allopurinol was not given in the acute flare of gout. The patient’s pain improved, and the range of motion of the right knee improved to 0° to 160°. He was discharged uneventfully after two days of hospitalisation.

## Discussion

Gout and pseudogout occurring in the same joint in a patient is rare. There is limited published data to suggest that these conditions can occur concomitantly^2-4^. Majority of the literature have described these cases occurring in the knee joint, but involvement of the ankle, shoulder and metacarpophalangeal joints have also been reported^4^. Stockman *et al* investigated patients with gout and found that there were concomitant radiographic features of pseudogout in 8 out of 138 (5.8%) patients in his series^3^. Whilst the exact pathology resulting in concomitant mono-sodium urate (MSU) and calcium pyrophosphate (CPP) crystal deposition is unclear, Ankli *et al* postulates that patients with long-term gout appear to have a higher risk of CPP co-deposition^4^. This is consistent with the clinical history in our patient, with recurrent episodes of gout flare potentiating the development of pseudogout.

Patients with acute gout or pseudogout flares often present with signs of inflammation such as knee effusion, pain and loss of range of motion^1^. Gross inspection of the knee aspirate may not effectively differentiate these diseases. Turbid fluid is a reflection of raised cell count and can be present in either gout or pseudogout^2^. Diagnosis of concomitant gout and pseudogout should be made with the aid of radiographs and polarised light microscopy. Some of the reports in the literature describing concomitant gout and pseudogout have utilised chondrocalcinosis to determine the presence of pseudogout^3^. However, in this case, we demonstrate that the absence of chondrocalcinosis does not eliminate the possibility of concomitant pseudogout disease.

Polarised light microscopy of synovial fluid is regarded as the gold standard to establish the presence of gout and pseudogout. Polarised light microscopy consists of a first-order red plate compensator, polariser filer and an analyser^2^. Manipulation of the slides alters the crystals’ orientation with the compensator axis and allows for differentiation of the crystals. MSU crystals appear yellow when oriented parallel to the compensator whilst CPP crystals appear blue when aligned with the compensator axis and show inclined extinction when misaligned with the axis of the polariser and analyser (weakly positive birefringence)^2^.

Despite the common utilisation of polarised light microscopy, there are technical challenges in accurate identification of crystals and microscopist competence is critical. Berendsen *et al* conducted an online test involving 110 professional rheumatologists, laboratory technicians and physicians^5^. Participants were made to identify slide images of different crystals. Only one in three participants was able to identify all the CPP crystals and one in twenty participants could not identify any typical picture of CPP at all. This study highlights the technical difficulty in diagnosing pseudogout under polarised light microscopy as both its shape and faint positive birefringence may be difficult to appreciate^5^. Given this finding, it may suggest an under-reporting of cases with concomitant gout and pseudogout due to diagnostic challenges.

It is thus important to be cognisant that these conditions can occur in tandem, even without radiographic changes or a positive clinical history of gout. In this case, the crystals present in the joint were minimal and did not appear together in a single microscopic field. The authors recommend that when assessing polarised light microscopy, one must be vigilant in actively looking out for the presence of the two types of crystals in patients, regardless of the history and lack of radiographic evidence of calcium pyrophosphate disease. Treatment of both gout and pseudogout is similar. The treatment remains largely targeted at gout and is effective in managing both conditions. Physiotherapy, non-steroidal anti-inflammatory medication and colchicine remain the mainstay of treatment.

In conclusion, gout and pseudogout can occur concomitantly in acute crystal arthropathies. Polarised light microscopy is a useful modality for diagnosis and physicians should actively exclude concomitant pseudogout, especially in patients suffering from recurrent attacks of gout.
